# Supplementary material for: ANGPTL2 expression in the intestinal stem cell niche controls epithelial regeneration and homeostasis
Source: EMBO J. 2017 Jan 2;36(4):409–24. doi: 10.15252/embj.201695690 (PMC5694950; doi:10.15252/embj.201695690)
Supplement: Supplementary file 1 — Appendix [file EMBJ-36-409-s001.pdf]

ANGPTL2 expression in the intestinal stem cell niche controls epithelial regeneration and homeostasis

Haruki Horiguchi, Motoyoshi Endo, Kohki Kawane, Tsuyoshi Kadomatsu, Kazutoyo Terada, Jun Morinaga, Kimi Araki, Keishi Miyata and Yuichi Oike

Appendix

Appendix Figure S1

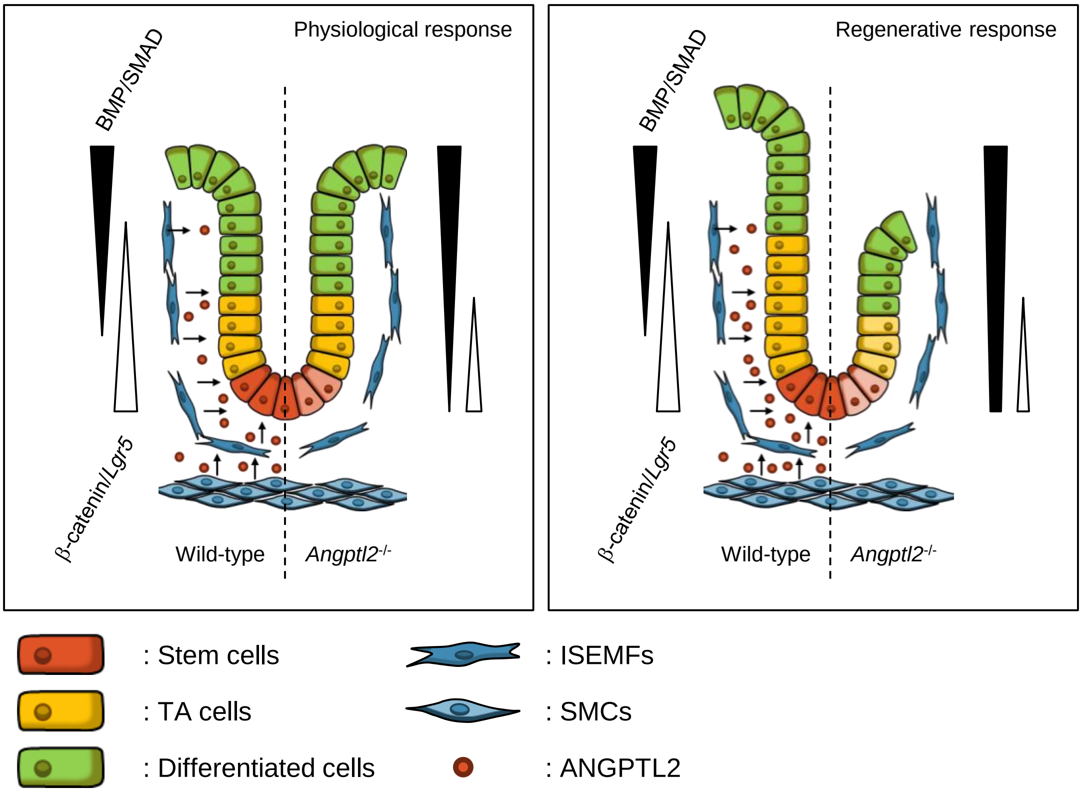

Appendix Figure S1. Effect of ISEMF-derived ANGPTL2 following structural or functional tissue damage

ISEMFs expressed higher levels of BMP in *Angptl2*$^{-/-}$ relative to wild-type mice, inactivating β-catenin signaling to decrease *Lgr5* mRNA induction in IECs. ANGPTL2 derived from ISEMFs maintained the ISC niche by modulating levels of competing signaling between BMP and β-catenin to maintenance ISCs.
