# Supplementary material for: ANGPTL2 expression in the intestinal stem cell niche controls epithelial regeneration and homeostasis
Source: EMBO J. 2017 Jan 2;36(4):409–24. doi: 10.15252/embj.201695690 (PMC5694950; doi:10.15252/embj.201695690)
Supplement: Supplementary file 2 — Expanded View Figures PDF [file EMBJ-36-409-s002.pdf]

Expanded View Figures

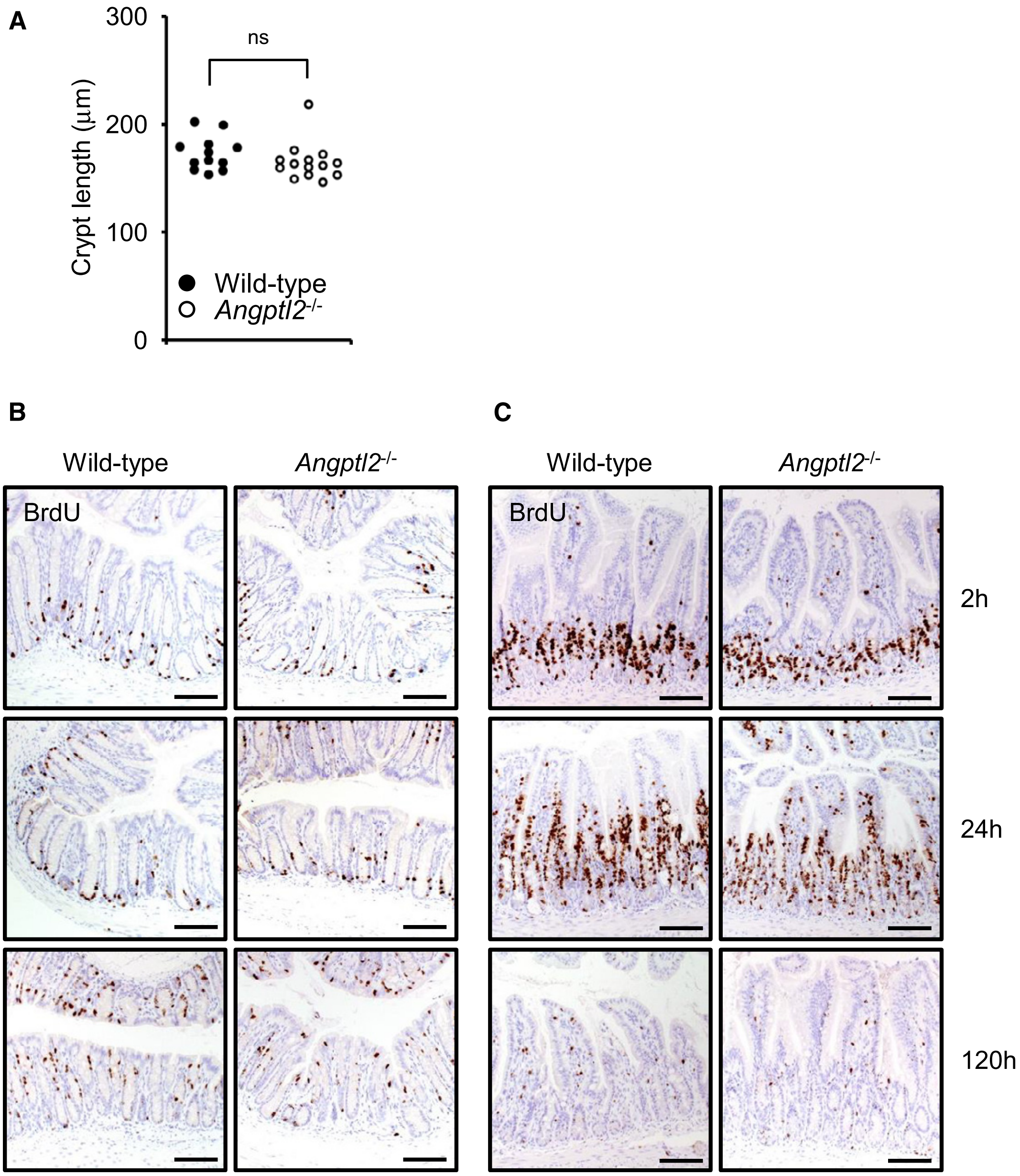

Figure EV1. ANGPTL2 is not required for normal intestinal development and does not appreciably regulate baseline epithelial cell turnover.

A Length of crypts in wild-type (n = 12) and *Angptl2*$^{-/-}$ (n = 14) mice. Average per mouse is shown as respective dots. ns, difference not statistically significant; unpaired Student's *t*-test.

B, C Representative images of BrdU IHC of colon (B) and small intestinal (C) epithelial cells at 2, 24, and 120 h after BrdU injection. Scale bar = 100 μm.

© 2017 The Authors

Figure EV2. ANGPTL2 functions in intestinal regeneration.

A *Angptl2* mRNA levels in wild-type mouse colon were determined following 6 days of DSS treatment (DSS) and then 6 more days with normal water. Controls were treated with normal water for all 12 days (Untreated). n = 4.

B Weight loss in wild-type (n = 10) and *Angptl2*$^{-/-}$ (n = 9) mice after initiation of 2.5% DSS treatment over the 12-day experiment.

C Overall disease activity of wild-type (n = 10) and *Angptl2*$^{-/-}$ (n = 9) mice administered 2.5% DSS in drinking water for 6 days and then untreated water for 6 more days.

D–F Representative images of colon crypts assessed by H&E (D), Ki67 (E), and pH3 (F) staining in wild-type and *Angptl2*$^{-/-}$ mice following DSS treatment (assessed at day 6). Scale bar = 100 μm.

G *Angptl2* mRNA levels in wild-type mice colon 5 days after 12-Gy irradiation (n = 5) compared to untreated controls (n = 4).

H, I Representative images of crypts of small intestine assessed by H&E (H) and Ki67 (I) staining in wild-type and *Angptl2*$^{-/-}$ mice 3 days after 12-Gy irradiation. Scale bar = 100 μm.

J, K Representative images of crypts of colon assessed by H&E (J) and Ki67 (K) staining in wild-type and *Angptl2*$^{-/-}$ mice 3 days after 12-Gy irradiation. Scale bar = 100 μm.

L Representative images of colons from wild-type and *Angptl2*$^{-/-}$ mice 5 days after 12-Gy irradiation.

Data information: Data are represented as mean ± SEM. *P < 0.05; **P < 0.01; ***P < 0.001. (A) Unpaired Student's t-test, (B) unpaired Student's t-test or Welch's t-test, (G) unpaired Welch's t-test.

 © 2017 The Authors

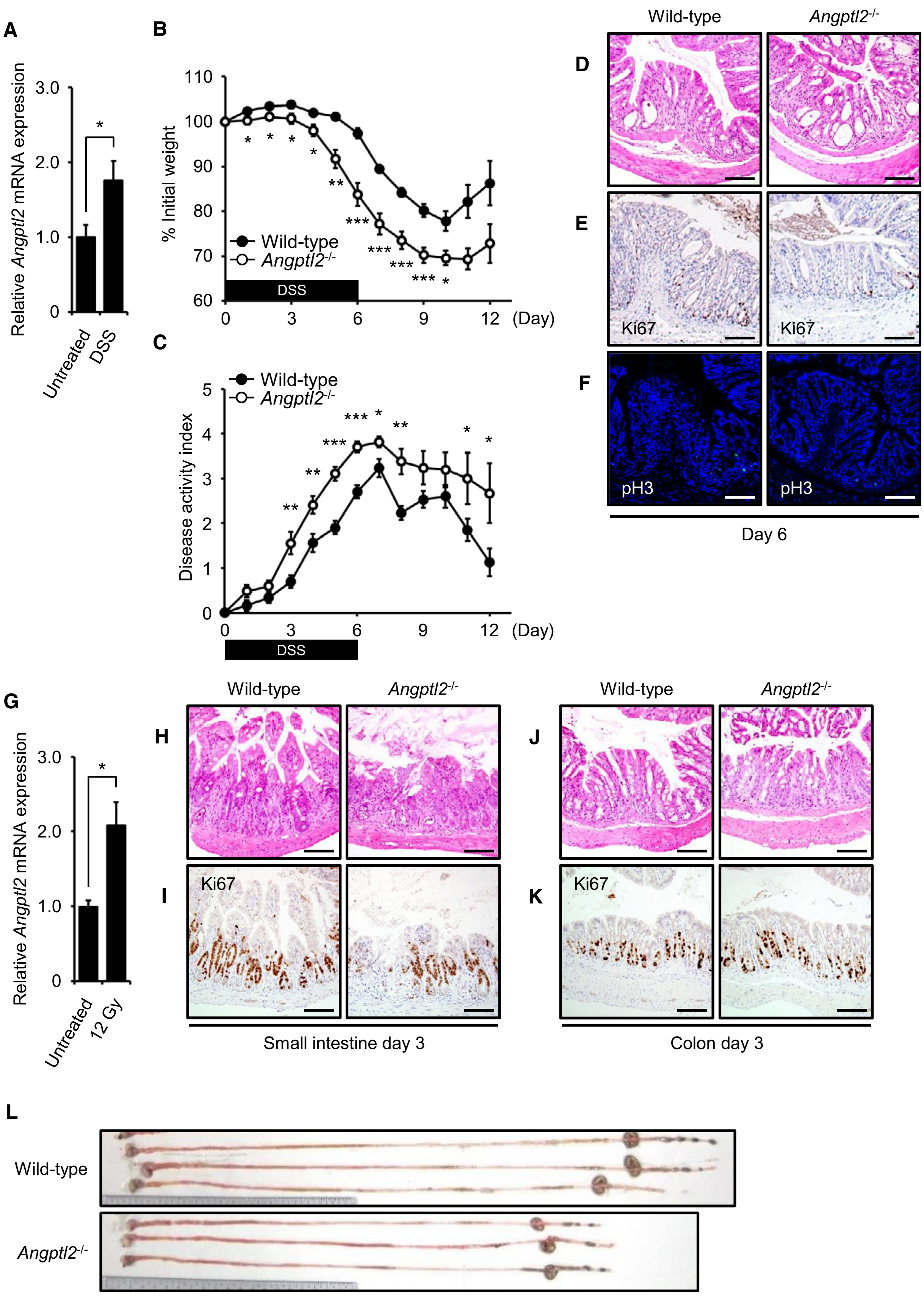
A
Relative Angptl2 mRNA expression
Untreated
DSS
B
% Initial weight
Wild-type
Angptl2-/-
DSS
(Day)
C
Disease activity index
D
E
F
Wild-type
Angptl2-/-
Ki67
pH3
Day 6
G
12 Gy
H
I
J
K
Small intestine day 3
Colon day 3
L

Figure EV2.

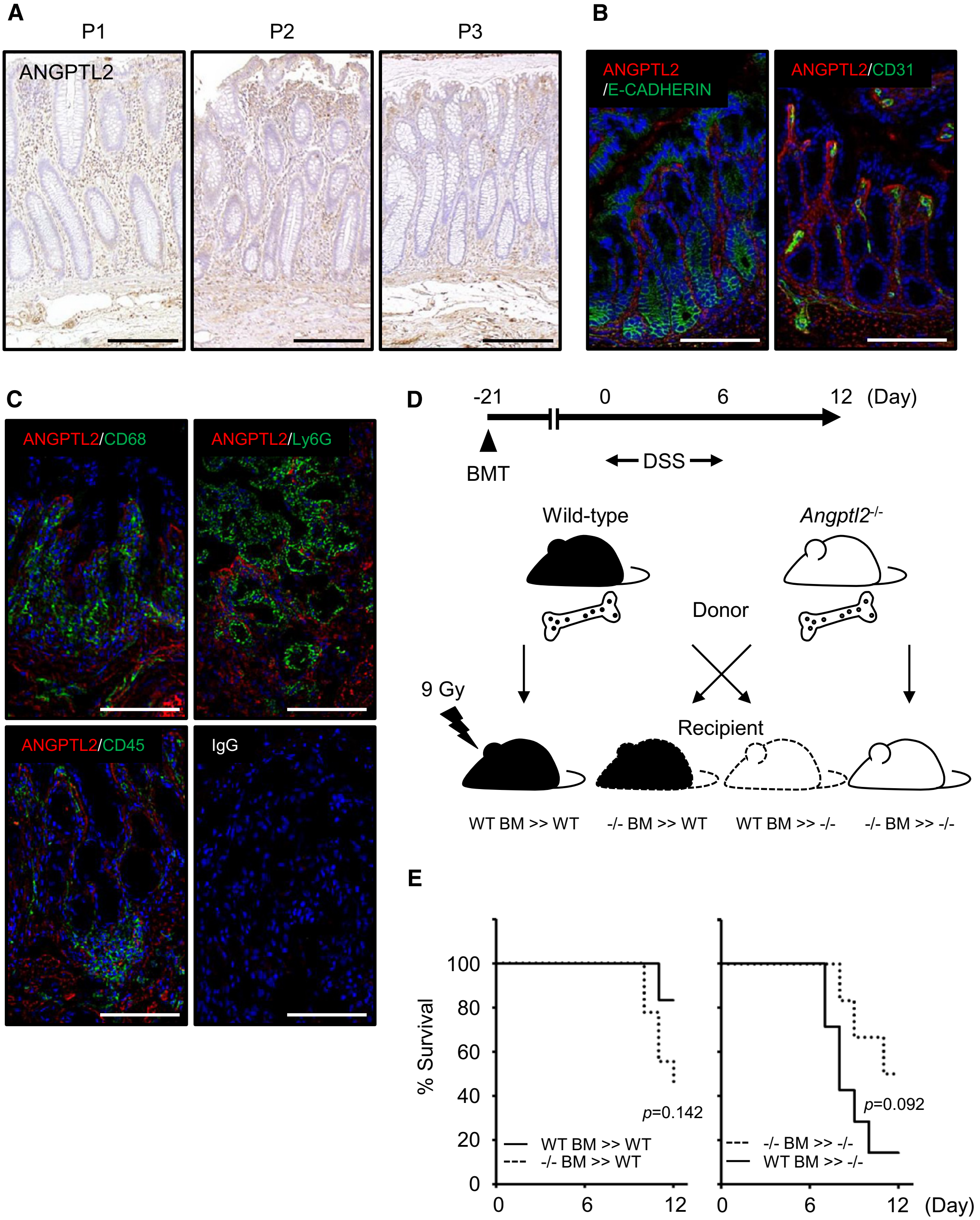

Figure EV3. ANGPTL2 localizes to the intestinal mesenchyme.

A IHC for ANGPTL2 in three samples of normal human colonic mucosa obtained from colectomy samples from subjects (P1, P2, and P3) with colorectal cancer. Scale bar = 100 μm.

B Representative IF images of distal colon tissues from wild-type mice analyzed with anti-ANGPTL2 (red) and anti-E-CADHERIN (green) or CD31 (green). Nuclei are counterstained with DAPI (blue). Scale bar = 100 μm.

C Representative IF images of distal colon tissue from wild-type mice following DSS treatment (assessed at day 6) evaluated with anti-ANGPTL2 (red) and anti-CD68, Ly6G, or CD45 (green). Nuclei are counterstained with DAPI (blue). Scale bar = 100 μm.

D Schematic showing relationships between donors and recipients in the BMT experiment. Mice were reconstituted with donor bone marrow for 21 days, and then, bowel inflammation was induced by 2.5% DSS in drinking water for 6 days followed by restoration of normal drinking water for 6 days thereafter.

E Survival rates of WT BM >> WT (n = 6), −/− BM >> WT (n = 9), −/− BM >> −/− (n = 6) and WT BM >> −/− (n = 7). P-values were calculated by log-rank test.

 © 2017 The Authors

Figure EV4. ANGPTL2 does not directly activate β-catenin signaling in epithelial cells *in vitro*.

A, B Experimental protocol of organoid regeneration assay.
C Dissociated organoid cultures treated with vehicle or rANGPTL2. Scale bar = 200 μm.
D Organoid growth efficiency and size after passage treated with vehicle or rANGPTL2 (n = 4). Data from vehicle were set at 1.
E Organoid cultures following 1-Gy irradiation and treated with vehicle or rANGPTL2. Scale bar = 200 μm.
F Organoid growth efficiency and size after 1-Gy irradiation and treated with vehicle or rANGPTL2 (n = 12). Data from vehicle were set at 1.
G mRNA levels of indicated transcripts in colon and epithelium from wild-type mice (n = 4) based on qRT–PCR analysis. *Intα5*, *integrin α5*; *Intβ1*, *integrin β1*.
H Integrin expression in HEK293, Caco-2, and SW480 cells. Representative profiles obtained by FACS analysis using indicated anti-integrin antibodies (black line traces) or control IgG (filled gray traces).
I Western blotting analysis of HEK293, Caco-2, and SW480 cells treated with vehicle or rANGPTL2. rA2, recombinant ANGPTL2. HSC70 serves as an internal control.

Data information: Data are represented as means ± SEM. ns, not statistically significant. (D) Unpaired Student's t-test, (F) unpaired Student's t-test.
Source data are available online for this figure.

© 2017 The Authors

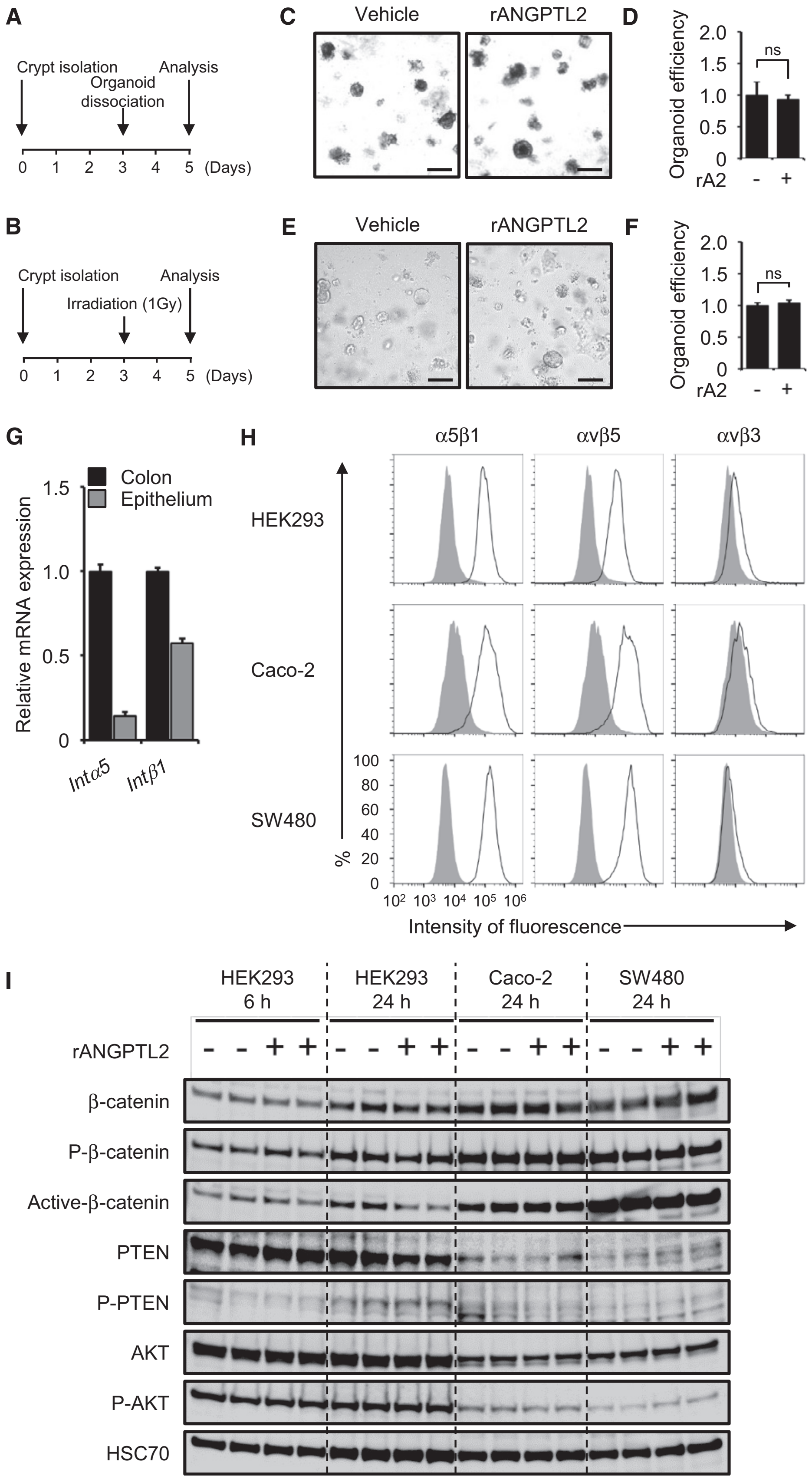

Figure EV4.

 © 2017 The Authors

Figure EV5. Organoid formation decreases following co-culture with *Angptl2*$^{-/-}$ ISEMFs or treatment with *Angptl2*$^{-/-}$ ISEMF CM.

A Schematic illustration of co-culture of colon organoids with ISEMFs separated by a Transwell membrane.
B Colon organoid cultures in the presence of ISEMFs with or without exogenous Noggin (Nog). Scale bar = 50 μm.
C Organoid growth efficiency and size in the presence of ISEMFs with or without exogenous Noggin (Nog). n = 4. Data from wild-type organoids/wild-type ISEMFs/Nog (+) were set at 1.
D Schematic illustration of colon organoid cultures in the presence of ISEMF CM.
E Organoid cultures in the presence of ISEMF and treated with or without Noggin (Nog). Scale bar = 50 μm.
F Growth efficiency and size of organoids treated with ISEMF CM, with or without Noggin (Nog). n = 4. Data from wild-type organoids/wild-type ISEMFs CM/Nog (+) were set at 1.

Data information: Data are represented as mean ± SEM. *$P < 0.05$; ns, not statistically significant. (C) Unpaired Student's *t*-test or Welch's *t*-test, (F) unpaired Student's *t*-test.

© 2017 The Authors

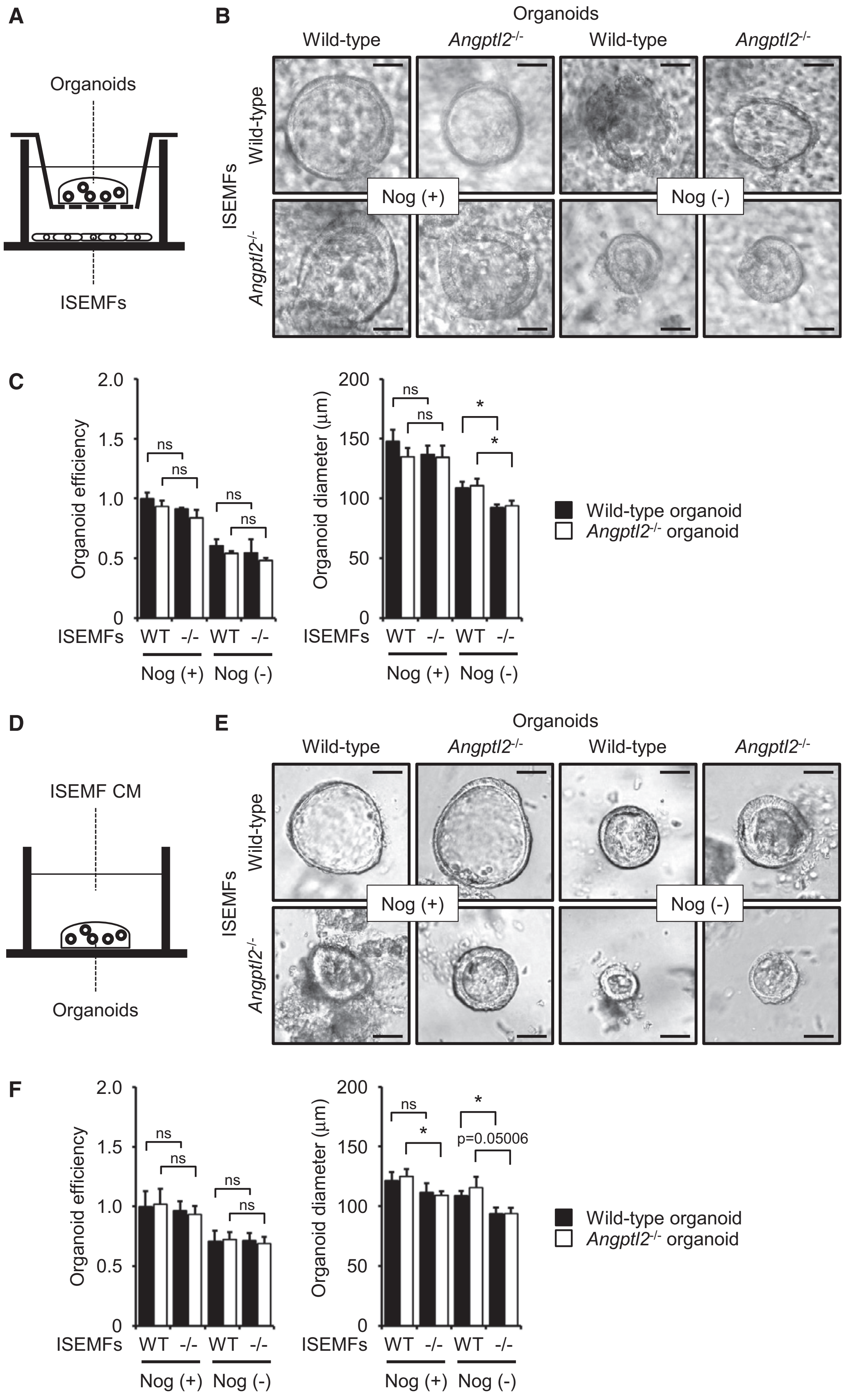

Figure EV5.

 © 2017 The Authors
