# Supplementary material for: ANGPTL2 expression in the intestinal stem cell niche controls epithelial regeneration and homeostasis
Source: EMBO J. 2017 Jan 2;36(4):409–24. doi: 10.15252/embj.201695690 (PMC5694950; doi:10.15252/embj.201695690)
Supplement: Supplementary file 3 — Table EV1 [file EMBJ-36-409-s003.docx]

**Table EV1**

| Scoring of disease activity index | | | |
| --- | --- | --- | --- |
| Score | Weight loss (%) | Stool consistency | Bleeding |
| 0 | None | Normal | Negative |
| 1 | 1-5 |  |  |
| 2 | 6-10 | Loose stool | Positive occult blood test |
| 3 | 11-20 |  |  |
| 4 | >20 | Diarrhea | Gross bleeding |
