# Supplementary material for: ANGPTL2 expression in the intestinal stem cell niche controls epithelial regeneration and homeostasis
Source: EMBO J. 2017 Jan 2;36(4):409–24. doi: 10.15252/embj.201695690 (PMC5694950; doi:10.15252/embj.201695690)
Supplement: Supplementary file 4 — Table EV2 [file EMBJ-36-409-s004.docx]

**Table EV2**

**Sequences of primers used in Real-time PCR analysis**

***Gene* Forward primer Reverse primer**

*mAngptl2* GGAGGTTGGACTGTCATCCAGAG GCCTTGGTTCGTCAGCCAGTA

*mE-cadherin* CGTCCTGCCAATCCTGATGA ACCACTGCCCTCGTAATCGAAC

*mSma* CCTGACGGGCAGGTGATC ATGAAAGATGGCTGGAAGAGAGTCT

*mVimentin* AAAGCGTGGCTGCCAAGAAC GTGACTGCACCTGTCTCCGGTA

*mWnt3a* AGGAGTGCCAGCACCAGTTC CATGGACAAAGGCTGACTCC

*mWnt2b* TGGATGCCAAAGAGAAGAGG ACTTGCACTCCAGCTTCAGG

*mRspondin1* CCTGGTTCTGAGCTGGACAC AACAGAGCTCACAGCCCTTG

*mBmp2* TGACTGGATCGTGGCACCTC CAGAGTCTGCACTATGGCATGGTTA

*mBmp4* AGCCGAGCCAACACTGTGAG TCACTGGTCCCTGGGATGTTC

*mBmp7*  ACATCCGGGAGCGATTTGAC TCCTCAGAAGCCCAGATGGTG

*mNoggin* CGGCCAGCACTATCTACACA GCGTCTCGTTCAGATCCTTC

*mGremlin1* ACTCGTCCACAGCGAAGAAC TCATTGTGCTGAGCCTTGTC

*mEgf* CCTCATATGATGGATACTGCCTCAA ACCAGTGCCACCATGCAGA

*mFgf* GGTTCAACCTGCCTCTAGGAAACT GCCGGTCTCCGTACCCTTTA

*mHgf* AGAAATGCAGTCAGCACCATCAAG GATGGCACATCCACGACCAG

*mMyc* GCTCGCCCAAATCCTGTACCT TCTCCACAGACACCACATCAATTTC

*mCyclind1* CATGTATCATCTAGCCATGCACGAG ATGCACAACAGGCCGCTACA

*mIntegrinα5* CAAGGTGACAGGACTCAGCA TGGTGTGGAGAGGTCTCTGG

*mIntegrinβ1* GGATTCTCCAGAAGGTGGCT CAAAGTGAAACCCAGCATCC

*mLgr5* TGCCCCGTGGCTTTCTTATC TTTCCCAGGCTGCCCATATC

*mAscl2* AAGCACACCTTGACTGGTACG AAGTGGACGTTTGCACCTTCA

*mIgf1* TCATGTCGTCTTCACACCTCTTCT CCACACACGAACTGAAGAGCAT

*mIgf2* ACAACTTCGATTTGAACCACATTC GAGAGCTCAAACCATGCAAACT

*mIgfbp5* TCAACGAAAAGAGCTACGGC GTCTCTTCAGCCATCTCGGA

*mIgfbp4* CTTCATCATCCCCATTCCAA AAGCTTCACCCCTGTCTTCC

*m18s* TTCTGGCCAACGGTCTAGACAAC CCAGTGGTCTTGGTGTGCTGA
