# Supplementary figures and images for: ANGPTL2 expression in the intestinal stem cell niche controls epithelial regeneration and homeostasis
Source: EMBO J. 2017 Jan 2;36(4):409–24. doi: 10.15252/embj.201695690 (PMC5694950; doi:10.15252/embj.201695690)

Source data for Figure EV4I

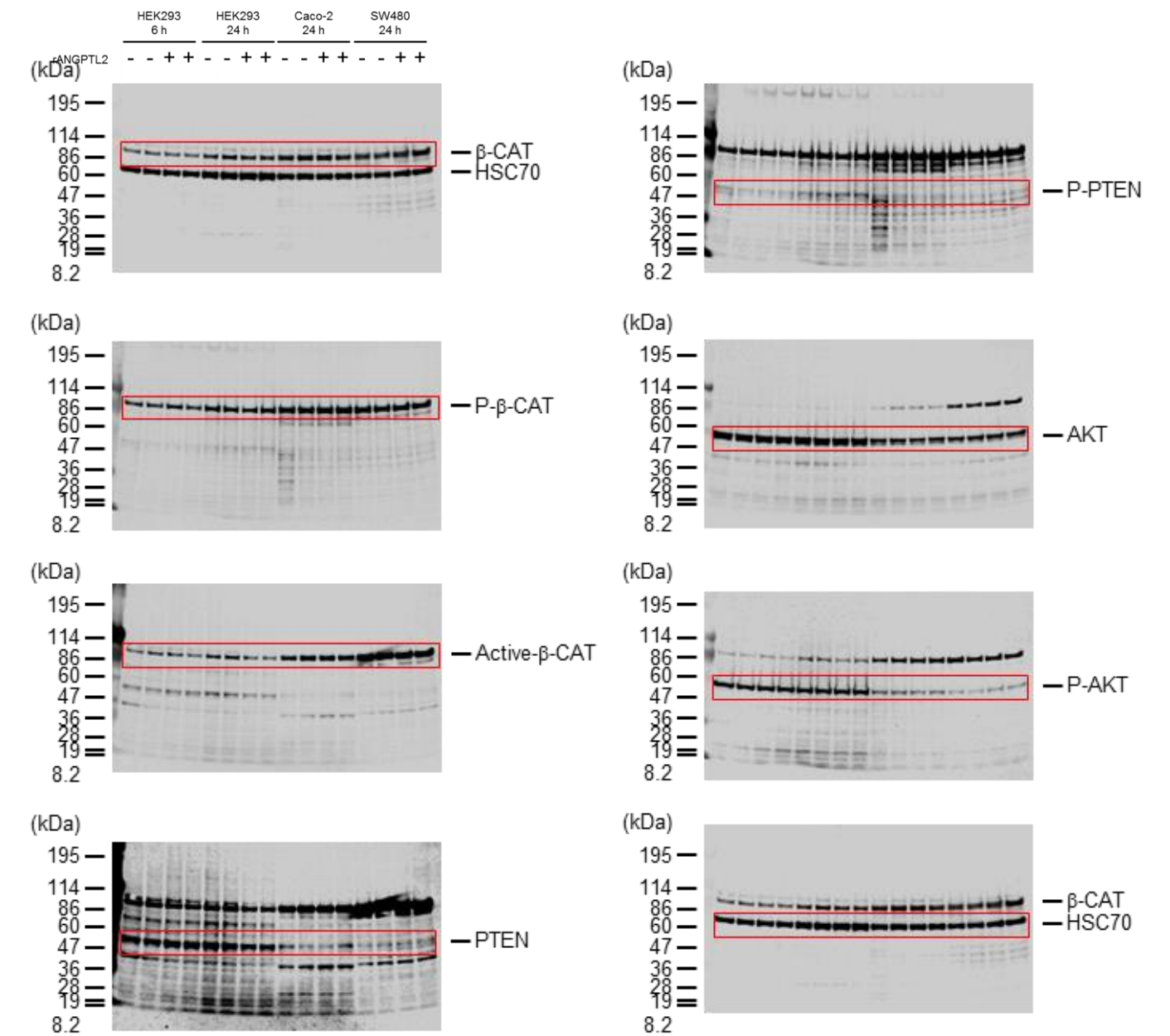

Supplement: Supplementary file 5 — Source Data for Expanded View [file EMBJ-36-409-s007.zip › EMBOJ_95690_EV4_source_data.pdf]

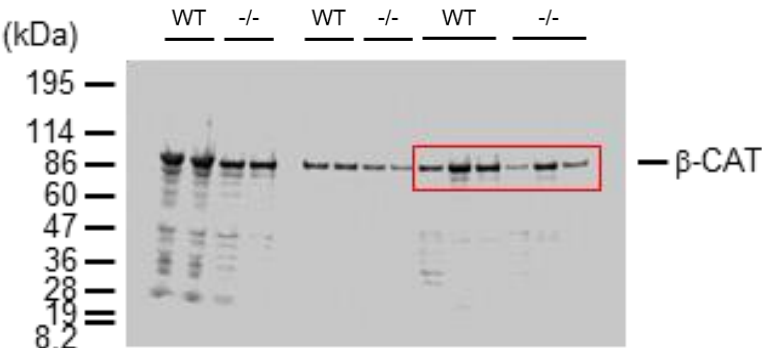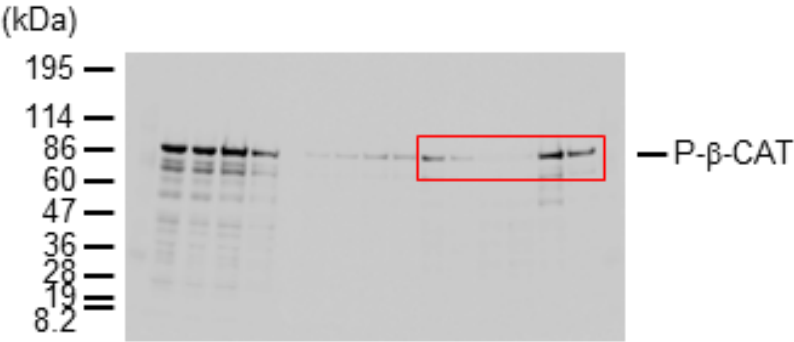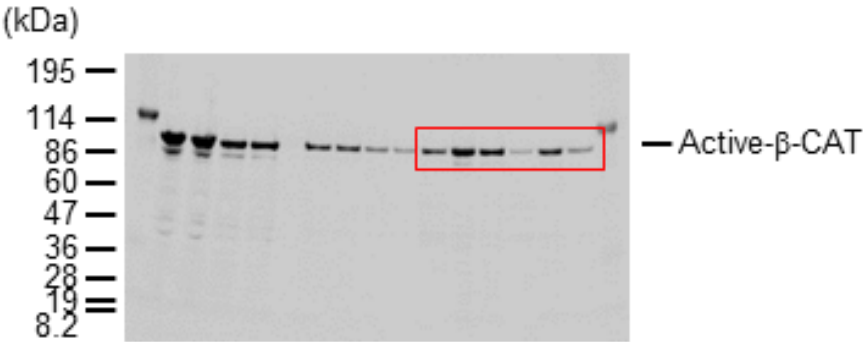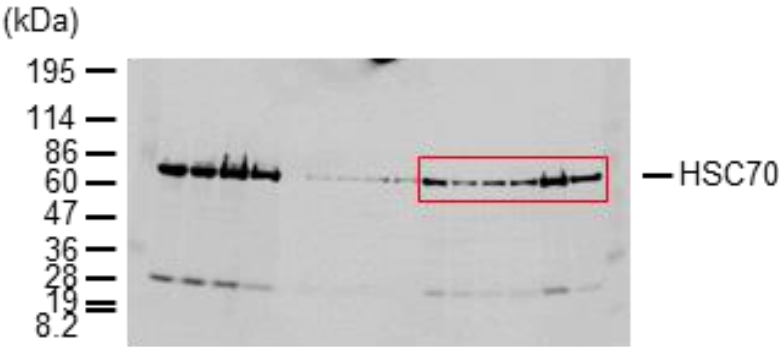

Supplement: Supplementary file 7 — Source Data for Figure 1 [file EMBJ-36-409-s005.pdf]

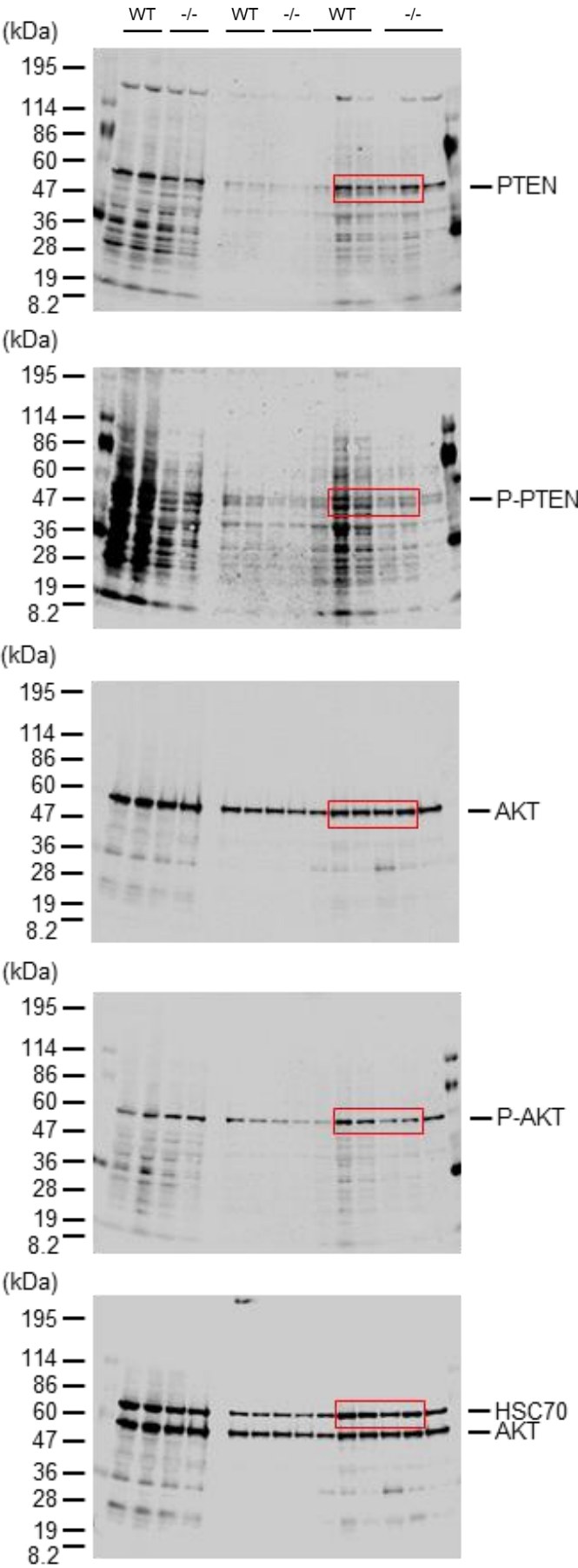

Supplement: Supplementary file 8 — Source Data for Figure 5 [file EMBJ-36-409-s006.pdf]
